# Both Viremia and Cytokine Levels Associate with the Lack of Severe Disease in Secondary Dengue 1 Infection among Adult Chinese Patients

**DOI:** 10.1371/journal.pone.0015631

**Published:** 2010-12-29

**Authors:** Yangbo Tang, Zhihua Kou, Fuchun Zhang, Xian Yao, Shengyong Liu, Jingming Ma, Yusen Zhou, Wei Zhao, Xiaoping Tang, Xia Jin

**Affiliations:** 1 Department of Laboratory Medicine, Guangzhou 8th People's Hospital, Guangzhou Medical College, Guangzhou, China; 2 State Key Laboratory of Pathogen and Biosecurity, Beijing Institute of Microbiology and Epidemiology, Beijing, China; 3 Department of Medicine, University of Rochester, Rochester, New York, United States of America; 4 Department of Biostatistics and Computational Biology, University of Rochester, Rochester, New York, United States of America; 5 Institute of Tropical Medicine, Southern Medical University, Guangzhou, China; University of Sao Paulo, Brazil

## Abstract

Secondary dengue infections are frequently associated with increased risk for dengue hemorrhagic fever and dengue shock syndrome. Surprisingly, we observed no dengue hemorrhagic fever cases among 353 hospitalized dengue-infected patients including 212 with primary, and 141 with secondary dengue 1 infection in China. To explore virological and immunological mechanisms which may account for this unexpected clinical observation, we assessed dengue viremia, type I interferon and inflammatory cytokine levels in these patients. While the levels of viremia and inflammatory cytokines are indistinguishable between primary and secondary infections, IFNα levels are significantly higher in primary than that in secondary infection. However, IFNα levels are positively correlated with dengue viremia levels (p<0.0001), but negatively correlated with the platelet counts (p<0.0001) and serum ALT levels (p = 0.0003). These results provide direct *in vivo* evidence that clinical dengue disease severity is affected by both viral and human immune factors.

## Introduction

Dengue viruses cause dengue fever in 50–100 million people annually, but only a small fraction of infections develop into dengue hemorrhagic fever and dengue shock syndrome (DHF/DSS). Some favor the idea that DHF/DSS are the result of immunopathology caused by cross-reactive responses of T cells or B cells [1], [2]; others place a greater emphasis on viral virulence factors [3]–[5]. It may be that both immune and viral factors affect the clinical outcome of dengue fever. Nonetheless, direct evidence on the relative contributions of immune and viral elements to dengue disease severity is lacking, especially in adult patients. In this study, we built on our previous observation that DHF/DSS cases were absent among 353 hospitalized Chinese patients with either primary or secondary dengue 1 infection (DENV1) [6] to explore the possible virological and immunological mechanisms that influence dengue disease severity.

Among immune factors, we focused on type I interferons, IFNα and IFNβ (IFNα/β), and a selective number of inflammatory and anti-inflammatory cytokines. IFNα/β are often secreted rapidly upon viral infection and provide the first-line of defense for uninfected cells through the induction of an antiviral state [7]–[9]. In patients with dengue virus infection, IFNα serum levels are elevated early [10], [11], suggesting it may contribute to virological control *in vivo*. In cell cultures *in vitro* and murine models *in vivo*, IFNα and IFNβ suppress early stages of dengue viral replication; whereas the type II interferon, IFNγ (produced mainly by activated T and NK cells), limits dengue viral replication at later stages [12]–[16]. IFNα and IFNβ can be induced by dengue virus infection in many cell types, but the kinetics of their induction differs. IFNβ gene activation is induced within hours of dengue viral infection in A549 lung carcinoma cell lines and Chang Liver cells [17]. In contrast, IFNα secretion is detected in dengue-infected primary human monocytes and plasmacytoid dendritic cells only 20 to 24 hours post virus inoculation [18]–[20]. While both IFNα and IFNβ have anti-dengue activity *in vitro*, previous clinical studies had limited to the measurement of IFNα levels, and most had been performed in subjects with either DENV2 or DENV3 infections [10], [11], [21]. In the current study, we performed, for the first time, a large-scale measurement of both IFNα and IFNβ in patients with DENV1 infection.

Cytokines that are undetectable in the blood of healthy individuals are elevated in dengue-infected patients, such as IL-1β, IL-2, IL-6, IL-8, IL-10, IL-13, IL-18, IFNγ, TNFα, and MCP1 [22]–[30]. Some of them are associated more closely with DHF/DSS than others. In comparison to those with uncomplicated dengue fever, patients with DHF/DSS have higher plasma levels of inflammatory cytokines IL-6, IL-8, and TNFα [25], [31]–[33], and both IL-8 and TNFα levels are independent correlates of dengue disease severity [24], [32]. The anti-inflammatory cytokine IL-10 has also been reported as having higher levels in DHF/DSS patients than patients with dengue fever [21], [29], [34]. In the current study, we chose to examine in detail the correlation between these host factors and viremia levels *in vivo*.

## Materials and Methods

### Study subjects and samples

The patient cohort has been described in details previously [6]. Briefly, sera or plasma samples were obtained from 353 hospitalized patients with acute DENV1 infection during a dengue outbreak in southern China in 2006. Dengue IgM and IgG capture ELISA kits (Panbio, Brisbane, Queensland, Australia) were used to diagnose primary and secondary dengue infections according to the manufacturer's protocol. Primary infection was diagnosed when only anti-dengue IgM was positive. Secondary dengue infection was diagnosed when both anti-dengue IgM and IgG were positive and IgM/IgG ratio<1.78, or when only IgG was positive and IgG level>22 units. By these diagnostic criteria, there were 212 patients with primary and 141 with secondary infections in our clinical cohort. Excluding those with HBV co-infections which account for about 8% of the total subjects [6], 170 patients with primary infection, and 115 patients with secondary infection were included in the current study. Sera from 37 healthy control adults were also collected. In 136 dengue-infected patients in whom extra serum samples were still available at the beginning of the current study, additional virological immunological analyses were performed. These studies have been approved by the Guangzhou 8^th^ People's Hospital Ethics Committee review. Written informed consents were obtained from all study subjects. National rules and regulations on human subject protection were strictly followed.

### Clinical and laboratory tests

Routine clinical tests including platelet count, liver function tests including aspartate aminotransferase (AST) and alanine transaminase (ALT) were performed as we previously described [6]. Serum interferon levels were measured using commercially available ELISA kits for IFNα (PBL InteferonSource, Piscataway, NJ) and IFNβ (Fujirebio Diagnostics, Inc., Malvern, PA) according to the manufacturer's instructions. The human IFNα multi-subtype ELISA kit detects 14 out of 15 known human IFNα subtypes (IFN-α1, IFN-α2, IFN-α4a, IFN-α4b, IFN-αA, IFN-αB2, IFN-αC, IFN-αD, IFN-αG, IFN-αH, IFN-αI, IFN-αJ1, IFN-αK, and IFN-αWA) with a lower limit of detection of 12.5 pg/ml. Human Interferon-β ELISA kit detects IFNβ with a lower limit of detection of 2.5 IU/ml. Serum cytokine levels for IL-6, IL-10, IFNγ, and TNFα were measured using commercially available ELISA kits (Diaclone, Besancon Cedex, France) according to the manufacturer's instructions.

### Quantification of viral RNA

Viral RNA was extracted from plasma sample using QIAamp Viral RNA Mini kit (QIAGEN) and transcribed into cDNA using TakaRa PrimeScript™ RT kit (TakaRa Biotech. Ltd., Da Lian city, China. www.takara.com.cn). Real-time PCR assay was then performed using TakaRa Premix Ex Taq™ (TakaRa Biotech. Ltd.) on an ABI7300 real-time PCR system (ABI, Foster city, CA). The primer and probe sequences were designed based on DENV1 isolated in Guangzhou (GenBank Accession #EU280167). The primers used were as follows: forward primer, 5′AAGGACTAGAGGTTAGAGGAGACCC3′ (nucleotides 10589 to 10613); reverse primer, 5′ CGTTCTGTGCCTGGAATGATG 3′ (nucleotides 10697 to 10677); and probe, FAM-5′ TCTGGTCTCTCCCAGCGTCAATATGCTGTT 3′-TAMRA (nucleotides 10657 to 10628) (where FAM is the reporter and TAMRA is the quencher). cDNA synthesis was performed in a total volume of 10µl containing 2µl of 5×PrimeScript buffer, 0.5µl of PrimeScript RT enzyme MixI, 2µl of random hexamer (100µM), 0.5µl of Oligo dT primer (50µM ), 2µl of viral RNA, and 3µl of RNase free dH_2_O. The RT conditions were 37° for 15m, followed by 85° for 5s. The PCR reaction was performed in a total volume of 20µl containing 10µl of 2×Premix Ex Taq, 0.4µl of forward primer (10µM), 0.4µl of reverse primer (10µM), 0.8µl of Probe (5µmol/L), 0.4µl of 50×ROX reference dye, 2µl of cDNA, and 6µl of RNase free dH_2_O. The PCR conditions were 95° for 30 s, followed by 40 cycles of 95° for 5s and 60° for 31s. A standard curve was established using a 10-fold serial dilution of plasmid DNA containing 10^3^ to 10^9^ copies of DENV1 genome. The lower limit of detection is 100 copies/ml. The viremia level of each patient sample was calculated according to the standard curve.

### Statistical analysis

Statistical analyses were performed using GraphPad Prism Version 4.0 (GraphPad Software, San Diego, CA). One-way analysis of variance (ANOVA) was used for mean comparison from multiple groups. Association between two laboratory outcomes was analyzed by Spearman rank correlation coefficient. A statistical test with P-value<0.05 was considered significant in mean comparison, or in correlation analysis.

## Results

### Clinical characteristics of patients with primary and secondary DENV1 infections

**Figure 1:**
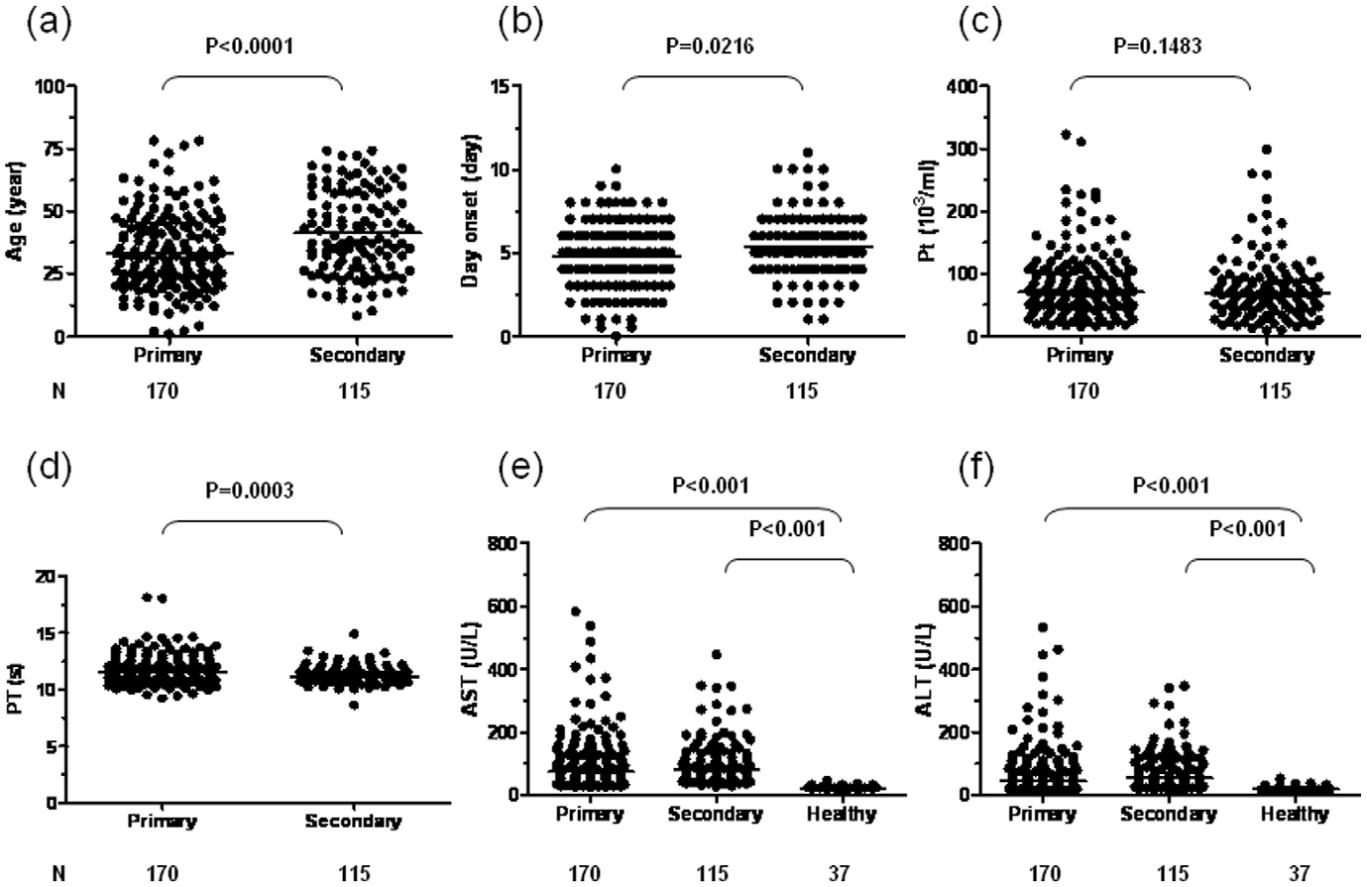
Demographic and clinical characteristics of patients with primary and secondary DENV1 infections. The age (a), fever onset day (b), platelet counts (c), PT (d), AST (e) and ALT levels (f) were summarized from 170 patients with primary and 115 patients with secondary DENV1 infections. Included also are AST and ALT levels from 37 healthy controls for comparison (e, f). Each dot represents one subject.

Excluding those with HBV-co-infections and those with incomplete clinical or laboratory data, 285 dengue-infected patients were included in this analysis. Among these patients, 170 and 115 had primary and secondary dengue infections, respectively. Key demographic characteristics of these two patient groups were summarized in **Figure 1**. Patients with secondary infection are older than those with primary infections (41.3±16.6 years vs. 33.2±15.6 years, p<0.0001, **Fig. 1a**) and have fever for a longer period of time (5.4±1.9 days vs. 4.8±1.8 days, p = 0.0216, **Fig. 1b**). This might due to the fact that adults usually do not go to hospital at the beginning of their illness, so samples were collected from them later than from younger patients, and older patients are more likely to have experienced dengue infection previously than younger patients. It is interesting to note that while the platelet counts were not different between the two groups (76.1±52.6×10^3^ /ml vs. 83.0±52.2×10^3^ /ml, p = 0.1483, **Fig. 1c**), the average prothrombin time (PT) was shorter in those with secondary infection (11.25±0.83 s vs.11.81±1.36 s, p = 0.0003, **Fig. 1d**). Both patient groups have higher AST (primary: 106.5±94.1 U/L; secondary: 104.1±76.0 U/L) and ALT (primary: 73.4±83.0 U/L; secondary: 77.6±65.6 U/L) levels than healthy uninfected controls (AST: 24.2±5.2 U/L; ALT: 20.6±8.2 U/L, p<0.001), but similar levels to each other (**Fig. 1e, f**).

### Immunological and viralogical features of patients with primary and secondary DENV1 infections

**Figure 2:**
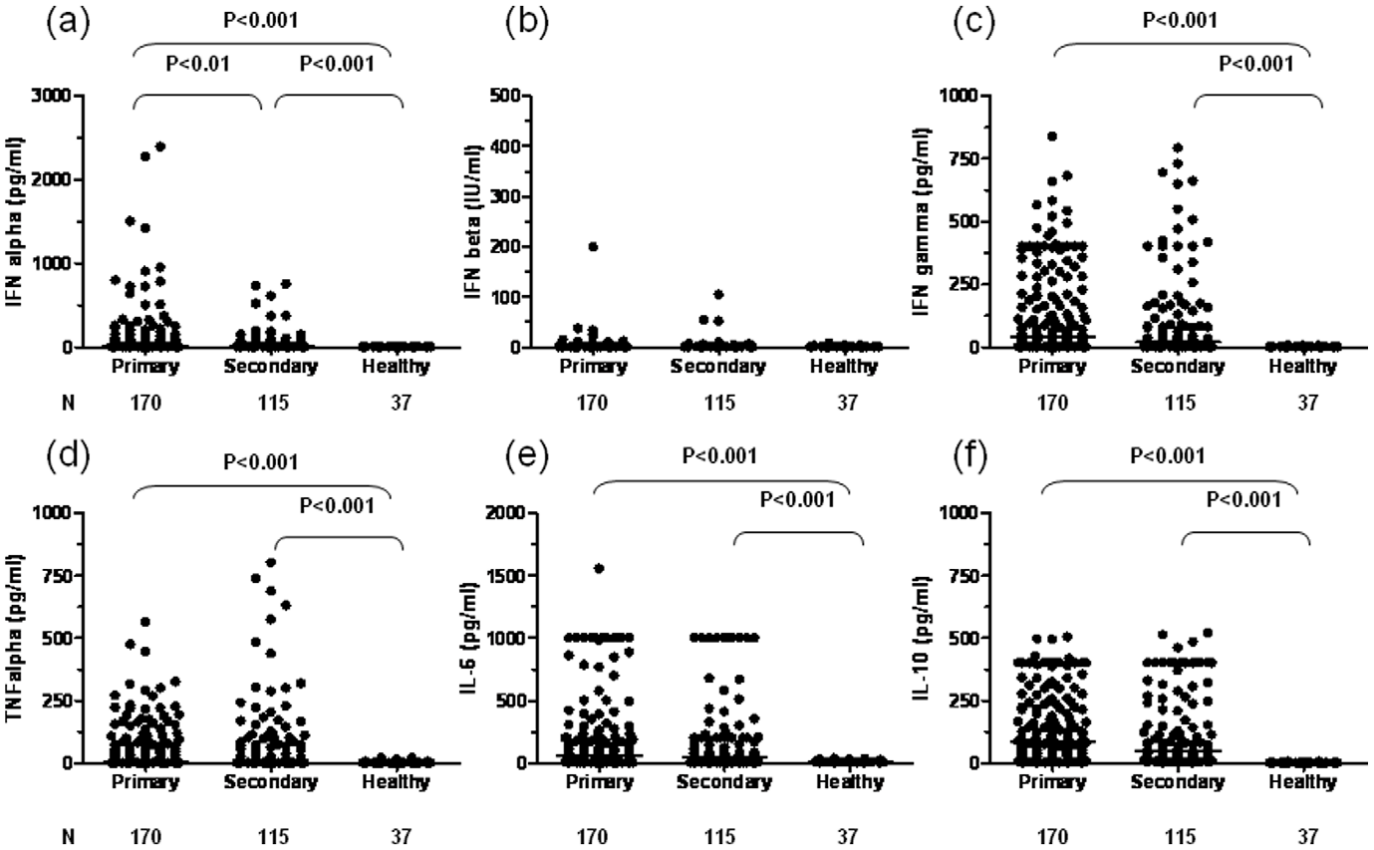
Distinctive patterns of cytokine production in patients with primary and secondary DENV1 infections. The serum levels of various cytokines including IFNα (a), IFNβ (b), IFNγ (c), TNFα (d), IL-6 (e), and IL-10 (f) from 170 patients with primary and 115 patients with secondary DENV1 infections and 37 healthy controls were presented. Each dot represents one subject. Except for IFNβ which was expressed as IU/ml, all other results were expressed as pg/ml.

To examine what else might be different between primary and secondary DENV1 infections, we next assessed the secretion of type I interferons and a selective number of inflammatory and anti-inflammatory cytokines in these patients. **Figure 2** shows that all dengue infected patients had secreted higher levels of IFNα than uninfected healthy controls (primary: 18.5 (0.0∼2392) pg/ml; secondary: 5.7 (0∼751.7) pg/ml, vs. control: 0 (0∼8.7) pg/ml, p<0.0001) and patients with primary infection had significantly higher IFNα levels than those with secondary infection (p<0.01) (**Fig. 2a**). In contrast, few infected subjects made substantial amounts of IFNβ (primary: 0.0(0∼199.2) IU/ml; secondary: 0.0(0.0∼104.3) IU/ml) and the IFNβ levels are not statistically higher than that in uninfected controls (control: 0.0(0.0∼158.4) IU/ml) (**Fig. 2b**). While patients with both primary and secondary infections produced large amounts of inflammatory cytokines including IFNγ, TNFα and IL-6 than uninfected controls (p<0.001), there are no differences between the two patient groups (**Fig. 2c, d, e** **)**; the same holds true for the anti-inflammatory cytokines, IL-10 (**Fig. 2f**).

**Figure 3:**
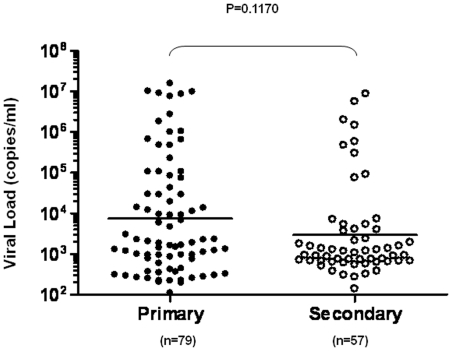
Lack of difference in viremia levels between patients with primary and secondary DENV1 infections. The plasma dengue viremia levels were measured in 79 patients with primary and 57 patients with secondary dengue infections using real-time qPCR as described in the Materials and Methods. Each dot represents one subject.

**Figure 4:**
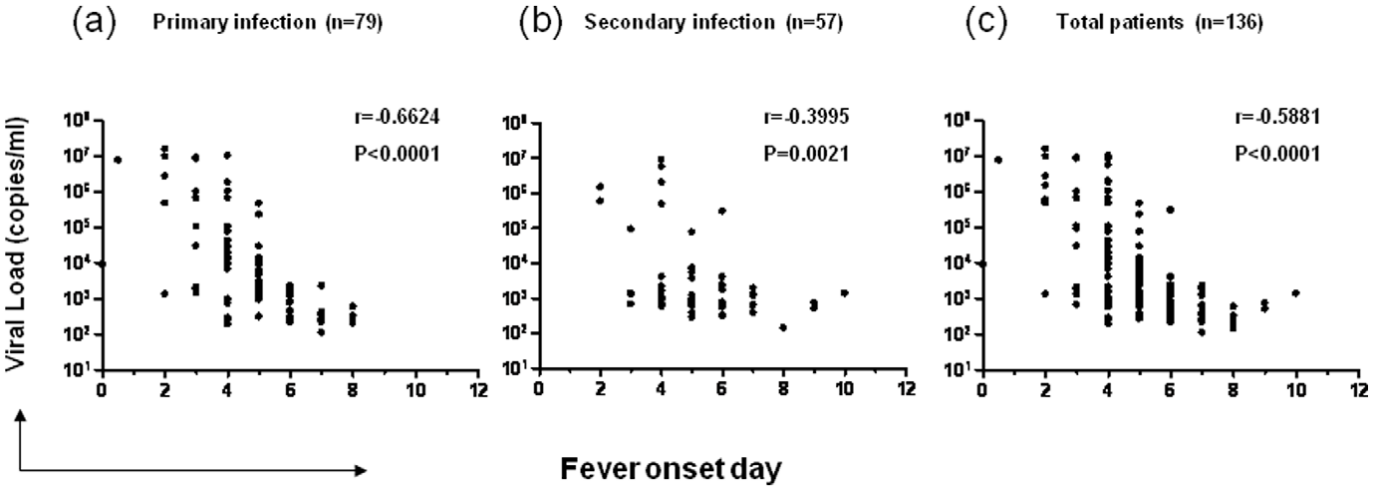
Rapid viremia decay in both primary and secondary DENV1 infections. The plasma dengue viremia levels over time were analyzed in 79 patients with primary (a) and 57 patients with secondary (b) dengue infections separately, or together (c). Each dot represents one subject. The fever onset day is defined as the duration from the time when patients first experienced fever symptom to the time when they were hospitalized and samples were collected.

In patients who had an additional vial of plasma sample available after all other clinical and immunological assays were performed, we also measured viremia levels using real-time quantitative PCR as described in the Materials and Methods. These include 79 patients with primary and 57 patients with secondary infection. Results show a lack of difference in viremia levels between the two patient groups (primary: 2.05×10^3^(1.10×10^2^∼1.57×10^7^) copies/ml; secondary: 1.08×10^3^(1.41×10^2^∼8.80×10^6^) copies/ml p = 0.1170, **Fig. 3**). Further analysis of the association between viremia levels and fever onset days (fever onset day is defined as the duration from the time when patients first experienced fever symptom to the time when they were hospitalized and samples were collected) showed that in both patient groups, viremia levels rapidly decreased within 8 days of fever onset when analyzed together (**Fig. 4c**) or separated into two subsets with either primary or secondary infections (**Fig. 4a, b**).

### IFNα levels are associated with multiple facets of dengue disease

**Figure 5:**
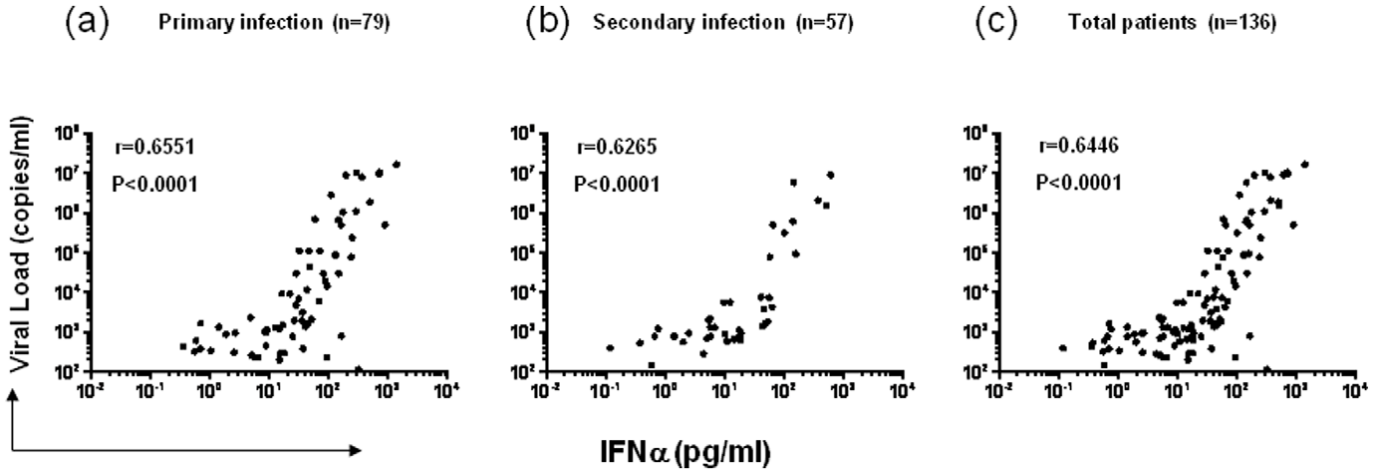
Positive correlation between viremia and IFNα levels in both primary and secondary DENV1 infections. The correlations between IFNα levels and plasma dengue viremia levels were analyzed in 79 patients with primary (a) and 57 patients with secondary (b) dengue infections separately, or together (c). Each dot represents one subject.

**Figure 6:**
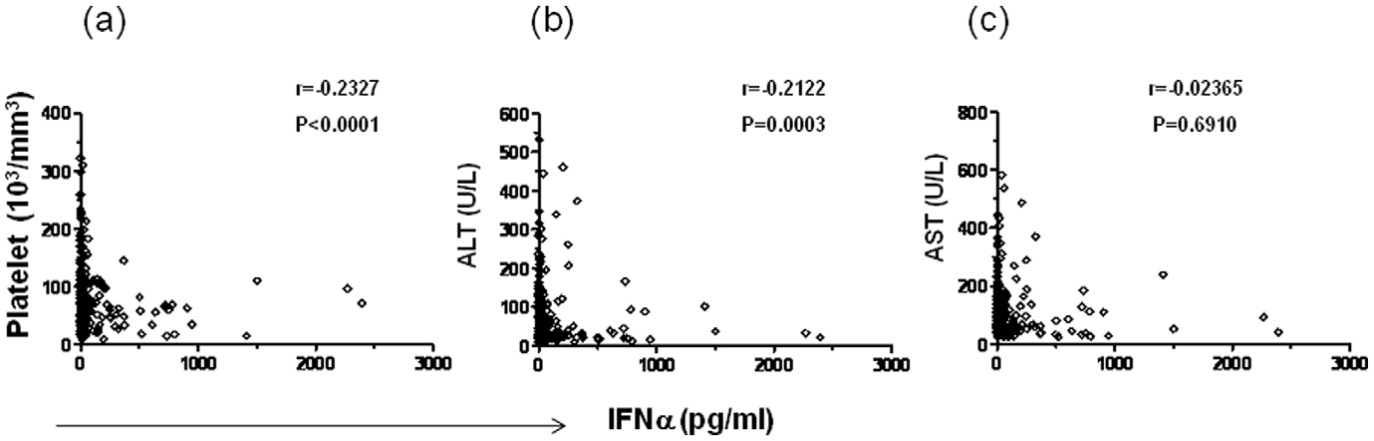
Levels of IFNα were negatively correlated with platelet counts and serum ALT levels. In the same cohort of 285 subjects with dengue fever due to either primary or secondary infection, the correlations between IFNα levels and platelet counts (a), ALT levels (b), and AST levels (c) were analyzed using Spearman rank correlation test. IFNα levels showed a significant negative correlation with platelet counts (r = −0.2327, P<0.0001) (**a**), and serum ALT levels (r = −0.2122, P = 0.0003) (**b**), but not serum AST levels (**c**). Each dot represents one subject.

To gain further insight into the interpretation of various clinical and laboratory measurements obtained, we performed Spearman correlation analysis on a number of parameters that could theoretically affect disease severity. Unexpectedly, we found viremia levels to be positively correlated with the levels of antiviral cytokine IFNα either for the entire group of 136 patients (r = 0.6446, p<0.0001, **Fig. 5c**), or two subsets with primary ( = 79) and secondary (n = 57) infections (**Fig. 5a, b**). This positive correlation becomes more significant when only those with viral load greater than 1,000 copies/ml were included in the analysis (primary = 51, secondary = 29, total = 80, r = 0.7733, p<0.0001, data not shown). Also unexpected was that the IFNα levels were negatively correlated with platelet counts (r = −0.2327, P<0.0001, **Fig. 6a**). In addition, IFNα levels were negatively correlated with serum ALT levels (r = −0.2112, P = 0.0003, **Fig. 6b**), but not with AST levels (**Fig. 6c**).

### Temporal secretion of IFNα, IFNγ, and IL-10 during dengue fever

**Figure 7:**
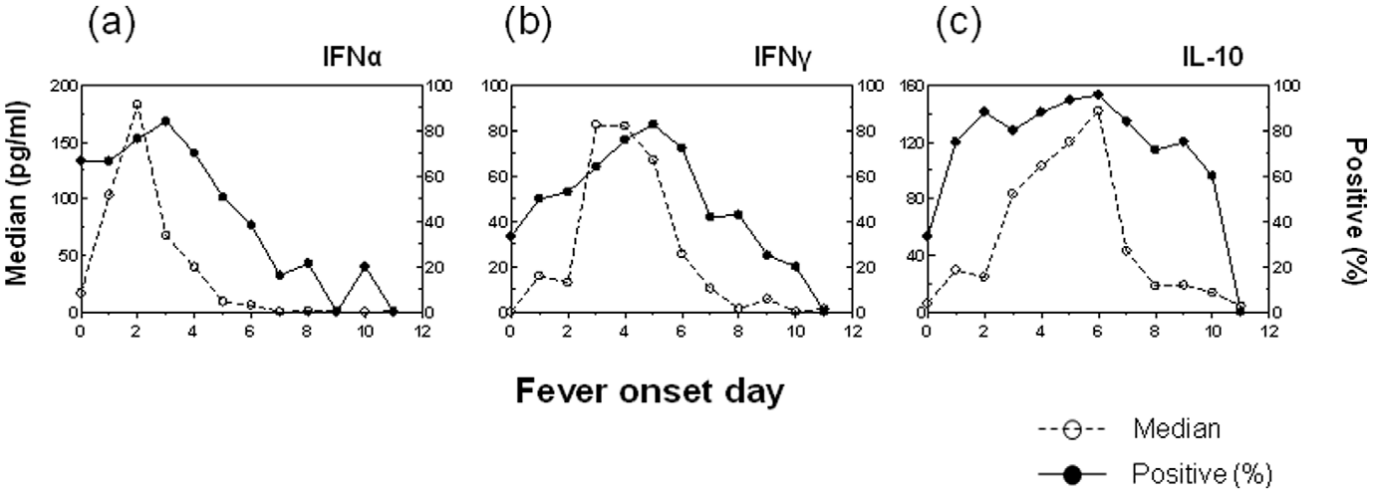
Temporal secretion of IFNα, IFNγ, and IL-10 during dengue fever. The associations between fever day and cytokine secretion including (a) IFNα, (b) IFNγ, and (c) IL-10 were analyzed in 285 subjects with dengue fever. The dashed line represents the median of cytokine levels, and the solid line indicates the percentage of subjects who had a positive cytokine level. Each symbol represents the median or average value at indicated time point.

We and other have found that many cytokines that are undetectable in the blood of healthy individuals are significantly elevated in dengue-infected patients [6], [22]–[30]. The interplay between type I interferons and these other cytokines may affect the clinical outcome of dengue disease. Because only IFNα was measurable at high levels in a large proportion of DENV1-infected subjects, and IFNβ was mostly undetectable, we next analyzed the kinetics of IFNα secretion in association with that of a pro-inflammatory cytokine, IFNγ, and an anti-inflammatory cytokine, IL-10. **Figure 7** shows a cross-sectional analysis between fever onset days and the median cytokine levels (left Y-axis), as well as the proportion of subjects who had a detectable level of cytokine responses in all 285 of DENV1-infected patients (right Y-axis). The median of IFNα level (182pg/ml) peaked on day 2 after fever onset and then decreased rapidly. Likewise, the percentage of patients who had low but detectable levels of IFNα (≥12.5pg/ml) was high on day 0 (within the first 24 hours of fever onset) (67%) and reached peak level on day 3 (84%) (**Fig. 7a**). The elevation of IFNγ levels followed the induction of IFNα and reached its peak level on days 3–4. In parallel, most subjects had detectable IFNγ on days 5–6 (**Fig. 7b**). In contrast, the IL-10 response only reached peak level by day 6 (142pg/ml), although a large proportion of subjects had detectable IL-10 secretion between days 2–6, an indication that IL-10 producers might be biologically more heterogeneous in the context of dengue viral infection (**Fig. 7c**). The same pattern of early IFNα secretion and late IL-10 production also holds true when similar analyses were performed by using patients with primary and secondary dengue infections separately (**Fig.S1**).

## Discussion

Both IFNα and IFNβ have anti-dengue activity in cell cultures *in vitro* and in animal models in *vivo*. However, previous clinical studies have only examined IFNα levels in the context of either DENV2 or DENV3 infections [10], [11], [21]. In the current study, by carefully measuring both IFNα and IFNβ levels in a large cohort of DENV1-infected adult patients, we found, for the first time, that IFNα levels are more significantly elevated than IFNβ during dengue fever. Moreover, in individuals with DENV1 infection, the IFNα elevation appears early, followed by the induction of a pro-inflammatory cytokine IFNγ, and lastly an anti-inflammatory cytokine IL-10. Unexpectedly, the levels of IFNα are directly proportional to viremia levels and the degree of thrombocytopenia.

A differential induction of IFNα and IFNβ in patients with dengue fever has never been reported before. This phenomenon is similar to that of acute HIV infection during which IFNα but not IFNβ level was rapidly, and transiently, elevated [35]. The exact reason for why only IFNα was made substantially in dengue and HIV-infected patients is not known, but it may be related to the fact that IFNα and IFNβ are produced by different cell types. IFNα is mainly produced by lymphoid cells, while IFNβ is made primarily by non-lymphoid cells. Since the principal dengue target cells are monocytic cells including monocyte, macrophage and dendritic cells [36]–[38] and HIV infects macrophage and CD4+ T cells, it may be expected that dengue and HIV infections directly stimulate relevant human cells to make IFNα, but not IFNβ. Nonetheless, IFNβ may be secreted subsequent to immune activation through either IFNα or other yet to be identified factors during viral infections. It is interesting to note that the temporal sequence of cytokine secretion is also similar between acute dengue and acute HIV infections, in that IFNα is made first, followed by the secretion of inflammatory cytokines, and lastly the anti-inflammatory cytokine IL-10 [35].

A significant increase in IFNα levels may contribute to the efficient control of dengue virus replication, but may also exert undesirable immune-regulatory effects on successive immune responses and thus play a more complex role in the pathogenesis of dengue fever. Unexpectedly, we observed that levels of IFNα were negatively correlated with platelet counts but positively correlated with viremia levels. These results suggest that elevated IFNα might be one of the factors contributing to decreased platelet counts during the course of the dengue fever. The regulation of platelet count during dengue virus infection is clearly due to multiple factors. For instance, it has been reported that IFNα2b could induce thrombocytopenia through the inhibition of platelet production from human megakaryocytes [39]. Our current finding may help to explain why patients with DHF have higher levels of IFNα than those with dengue fever [10]. In this regard, it is worth noting that persistent activation of IFNα gene in human peripheral blood mononuclear cells by another arbovirus, chikungunya, correlates with chronic disease [40], [41], and plasma viral loads and IFNα levels are directly proportional in chikungunya virus infected adults [42].

It is paradoxical that antiviaral IFNα levels shoud have a postitive correlation with viremia levels *in vivo*. This highlights the intricate interactions between a host immune factor and dengue viral replication. Although the precise reason for this is unclear, the time of sample collection may determine what type of correlations will be observed. In an experimental dengue infection system, we detected IFNα/β secretion immediately after virus production in DENV2-infected primary human monocytes, however, the kinetics of IFNα/β secretion was temporally associated with the decrease of virus production over a 5 day period (Kou, Virology, in press). A similar temporal association between viremia and IFNα secretion has also been reported in DENV3-infected Thai children [11]. Thus, our data offer a snap-shot of the dynamic interactions between dengue viral replicaiton and IFNα-mediated host defense mechanisms, and should not be interpreted as IFNα does not control virus replication.

It is interesting to note that IFNγ, IL-6 and IL-8 levels were found to be higher in secondary than primary infections in a recent study of dengue-infected patients in Western India [43]. In our current study, none of the cytokines measured including IFNγ, TNFα, IL-6 and IL-10 were statistically significantly different between patients with primary and secondary infections. The contrasting results may be explained by variability in dengue serotypes and the timing of sample collection. Our patients were homogeneous with DENV-1 infection and without DHF, whereas their patients were infected by all 4 DENV serotypes and a third of whom had DHF. Additionally, a large number of patients in our cohort had cytokine levels in the range of hundreds to thousands (pg/ml) because we enrolled most patients early after the onset of symptoms, whereas the majority of their patients had cytokine levels in the tens and a few of them in the hundreds (pg/ml) because nearly half of their patients were recruited late after the onset of illness [43]. Moreover, it is possible that the genetic differences between the Indian and Chinese populations have contributed to the observed discrepancy between these two studies.

In summary, we compared the levels of viremia, IFNα/β, and a selective number of cytokines in a large cohort of acutely DENV1 infected patients with either primary or secondary infection. Our results suggest that a lack of severe disease in this cohort of secondary DENV1 infected adults could be explained in part by both immune and virologic factors. It may also be possible that unique viral factors such as virus strain or genetic factors such as HLA alleles in the population could affect clinical presentation significantly. These should be focus of future studies. Overall, our findings not only advanced the understanding of human dengue pathophysiology, but also had implication in developing new anti-dengue therapeutics.

## Supporting Information

Figure S1 Temporal secretion of IFNα, IFNγ, and IL-10 in patients with dengue fever. The associations between fever day and cytokine secretion including IFNα (a, b) IFNγ (d, e), and IL-10 (g, h) were analyzed in either in 170 subjects with primary dengue infection (a, d, g), in 115 subjects with scondary dengue infection (b, e, h). The dashed line represents the median of cytokine levels, and the solid line indicates the percentage of subjects who had a detectable cytokine level. Each symbol represents the median or average value at indicated time point.
(TIF)
