# Supplementary figures and images for: Both Viremia and Cytokine Levels Associate with the Lack of Severe Disease in Secondary Dengue 1 Infection among Adult Chinese Patients
Source: PLoS One. 2010 Dec 29;5(12):e15631. doi: 10.1371/journal.pone.0015631 (PMC3012067; doi:10.1371/journal.pone.0015631)

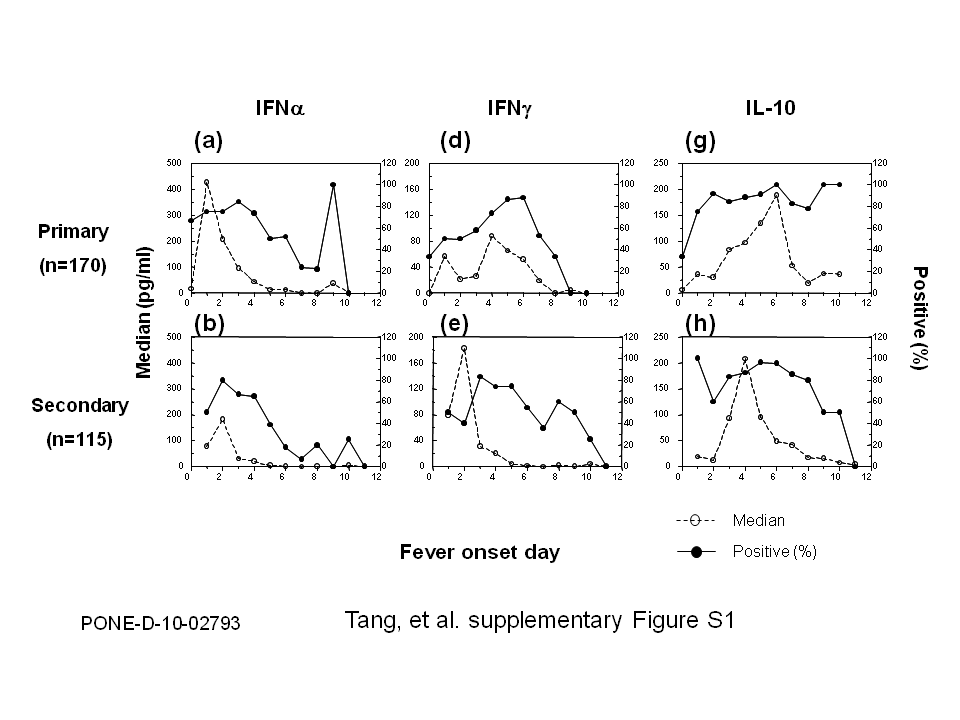

Supplement: Figure S1 — Temporal secretion of IFNα, IFNγ, and IL-10 in patients with dengue fever. The associations between fever day and cytokine secretion including IFNα (a, b) IFNγ (d, e), and IL-10 (g, h) were analyzed in either in 170 subjects with primary dengue infection (a, d, g), in 115 subjects with scondary dengue infection (b, e, h). The dashed line represents the median of cytokine levels, and the solid line indicates the percentage of subjects who had a detectable cytokine level. Each symbol represents the median or average value at indicated time point. (TIF) [file pone.0015631.s001.tif]
